# Shift in the function of netrin-1 from axon outgrowth to axon branching in developing cerebral cortical neurons

**DOI:** 10.1186/s12868-017-0392-x

**Published:** 2017-10-17

**Authors:** Hideko Matsumoto, Masabumi Nagashima

**Affiliations:** Department of Anatomy, Faculty of Medicine, Saitama Medical University, 38 Morohongo, Moroyama-machi, Iruma-gun, Saitama 350-0495 Japan

**Keywords:** Cortical development, Axon guidance, Axon outgrowth, Axon collateral branching, Netrin-1, Atmospheric scanning electron microscopy

## Abstract

**Background:**

Netrin-1, a multifunctional axon guidance cue, elicits axon outgrowth via one of its receptors deleted in colorectal cancer (DCC) in several types of neurons, including cerebral cortical neurons of embryonic mice. However, we and others have observed de novo formation of axon branches without axon outgrowth induced by netrin-1 in cortical culture of neonatal hamsters. These previous reports suggested the possibility that netrin-1 function might alter during development, which we here investigated using dissociated culture prepared from cerebral cortices of embryonic mice.

**Results:**

Imaging analysis revealed netrin-1-induced outgrowth in embryonic day (E) 14 axons and netrin-1-induced branching in E16 axons. Netrin-1-evoked filopodial protrusions, which sprouted on the shafts of E16 axons preceding branch formation, were visualized by a novel method called atmospheric scanning electron microscopy. Treatment with an anti-DCC function-blocking antibody affected both axon outgrowth and branching.

**Conclusions:**

Morphological analyses suggested a possibility of a shift in the function of netrin-1 in cortical axons during development, from promotion of outgrowth to promotion of branch formation starting with filopodial protrusion. Function-blocking experiments suggested that DCC may contribute not only to axon outgrowth but branching.

## Background

Netrin-1 is known as a diffusible axon guidance cue that plays various important roles in the correct wiring of the nervous system during development. Netrin-1 induces axon outgrowth (which is closely related to growth cone attraction) via one of its receptors DCC (deleted in colorectal cancer) in several types of neurons [1–4], including cerebral cortical cultures prepared from embryonic rats [5, 6] and embryonic day (E) 12.5 mice [7]. However, we and others have observed netrin-1-induced axon branching without axon outgrowth in cerebral cortical neurons prepared from the neonates of Syrian golden hamster, *Mesocricetus auratus* [8–11]. More recently, netrin-1-induced axon branching was also reported in cortical neurons prepared from E15 [12] and E15.5 [13] mice. Evidence has been accumulated indicating that axon branching is modulated independently of outgrowth, thus branches can form and extend toward targets while their primary axons stall or retract [10, 14]. This has been suggested to hold true for netrin-1-dependent axon branching [9, 10, 15].

Although less understood than axon outgrowth, two distinct modes are known for axon branching: “growth cone bifurcation” and “axon collateral branching” [16]. It has been reported that netrin-1-dependent axon branching takes place through the latter mode (also referred to as “de novo formation of axon branches” or “interstitial branching”), where netrin-1 first induces sprouting of filopodial protrusions from the axon shafts and then only part of the protrusions develop into axon branches [9, 15]. In the case that an actin filament-based filopodial protrusion develops into a collateral branch without being retracted back into the axon, invasion of axonal microtubules into the protrusion is observed [16, 17].

Neonates of the hamster are thought to be less mature than those of other rodents, reflecting its shorter (16-day) gestation period [15, 18, 19]. Indeed in hamsters, corticospinal fibers from motor cortex are just beginning their outgrowth at birth and thus the corticospinal pathway develops entirely postnatally [18], while corticospinal fibers of rats have already reached the cervical spinal cord on the day of birth [20]. Thereafter, in hamsters, the corticospinal axons reach pyramidal decussation in the caudal medulla on postnatal day (P) 3 [18], whereas in mice, late P0/early P1 is the timing when they reach pyramidal decussation and begin to form collateral branches to basilar pons (P0 = the first 24 h after birth) [21]. Studies with mutant mice revealed that netrin-1 signaling via DCC, as well as via another type of netrin receptor UNC5 that mediates repulsion, is involved in midline crossing at the pyramidal decussation [22].

Collectively, these previous reports raised the possibility of a shift in the function of netrin-1 in rodent cortical neurons during development, from promotion of axon outgrowth to promotion of axon collateral branching. In the present study we investigated this possibility by morphometric imaging analysis employing dissociated culture of cerebral cortical neurons prepared from mouse embryos.

Corticofugal tracts are comprised of subcerebral axonal tracts (including the corticospinal tract) and the corticothalamic projection. Embryonic and early postnatal development of those in mice has been studied in detail [7, 22–24], and following events are among those that have been reported to occur during mid to late embryonic development in mice. In E12.5 embryos, corticofugal axons have started to extend in the intermediate zone of the cortical wall toward ganglionic eminence (through which the internal capsule travels), which serves as an intermediate target by secreting netrin-1, providing a chemoattractive gradient for these axons. From E13.5 to E14.5, corticospinal axons (generated by neurons which reside in cortical layer 5) make a ventromedial turn out of the intermediate zone and enter the internal capsule, and their growth into the internal capsule is regulated by netrin-1/DCC; thereafter, these axons pass through the cerebral peduncles, and reach the upper level of the brainstem by E17. On the other hand, corticothalamic axons (mainly generated by neurons which reside in cortical layer 6) extend through the intermediate zone to reach the lateral internal capsule between E13 and E15.5, where they briefly pause until they resume extension within E15.5; between E16.5 and E17.5, they continue to extend in the internal capsule to approach the lateral border of the ventral thalamus, where they pause again. In the present study, dissociated cortical neurons prepared from E14 and E16 mice were subjected to the analysis.

We also performed visualization of netrin-1-dependent filopodial protrusions on the axon shafts which were thought to be induced transiently in advance of branch formation, by a novel method called atmospheric scanning electron microscopy (ASEM) [25]; this technique enabled observation of samples in solution with resolution of electron microscope, thus unlike conventional electron microscopy, free from the artifacts caused by the pretreatments of specimens to put them into vacuum.

Furthermore, in this study we investigated whether the receptor DCC would be involved in the netrin-1 function of promoting branch formation in cortical axons, as other receptors such as UNC5 and DSCAM (Down syndrome cell adhesion molecule) are also known to mediate netrin signaling [26–28]. Contribution of DCC-mediated netrin signaling to arborization of retinal ganglion cell axons has been reported in the developing retinotectal system of frogs [29]. The results reported here suggest the possibility that DCC would contribute not only to axon outgrowth but also to de novo formation of axon branches in mammalian cerebral cortical neurons.

## Results

### Immunocytochemical detection of DCC in mouse primary cortical neurons

**Fig. 1:**
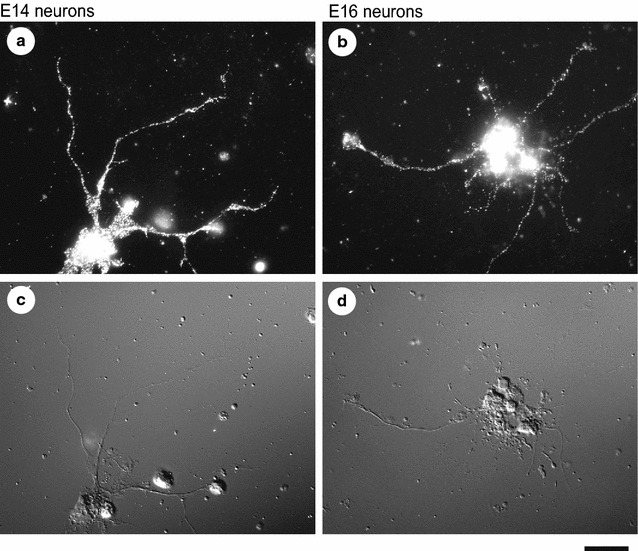
Expression of DCC protein in mouse primary cortical axons. Neurons prepared from E14 (**a**) and E16 (**b**) mice were fixed after 5 DIV, permeabilized, and then immunostained for DCC. DIC images of the same fields are also presented below (**c**, **d**). Scale bar, 20 μm

First, we carried out immunocytochemical analysis of DCC in cortical neurons prepared from E14 and E16 mice and grown for 5 days. Expression of DCC protein was observed both in E14 and E16 cortical axons (Fig. 1).

### Morphometric analysis of axon branching/outgrowth

**Fig. 2:**
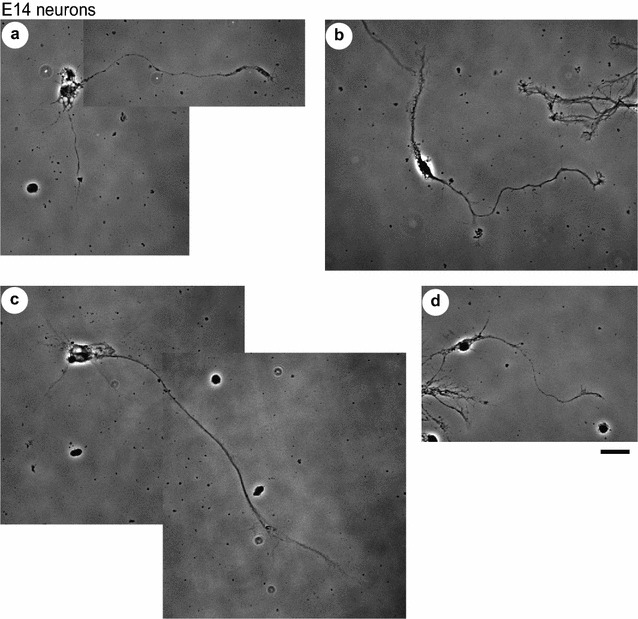
Netrin-1-induced axon outgrowth in mouse E14 cortical neurons. Phase contrast images shown are representative of E14 neurons under basal conditions with few protrusions (**a**) and abundant in protrusions (**b**) on the axon shafts, and those after netrin-1 stimulation in the absence (**c**) or presence (**d**) of an anti-DCC function-blocking antibody. Netrin-1 was bath-applied to cultures at a concentration of 250 ng/mL for 4 h, with or without 1 μg/mL anti-DCC antibody applied 30 min before the addition of netrin-1. Scale bar, 20 μm

**Fig. 3:**
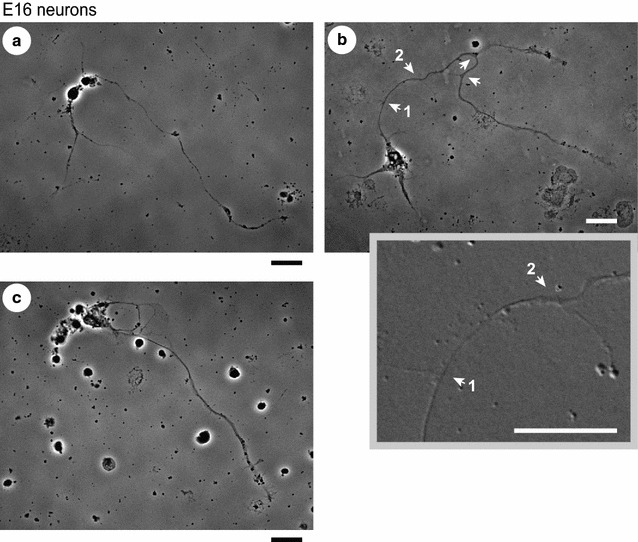
Netrin-1-induced axon branching in mouse E16 cortical neurons. Phase contrast images shown are representative of E16 neurons under basal conditions (**a**) and after 4 h netrin-1 stimulation (250 ng/mL) in the absence (**b**) or presence (**c**) of an anti-DCC function-blocking antibody (1 μg/mL; applied 30 min before the addition of netrin-1). Arrows indicate branch points (with branches longer than 12 μm and/or lamellipodium-tipped) along the primary axon. In **b**, an inset showing a DIC image provides a magnified view of the branch points indicated by the arrows No. 1 and No. 2. Scale bars, 20 μm

**Fig. 4:**
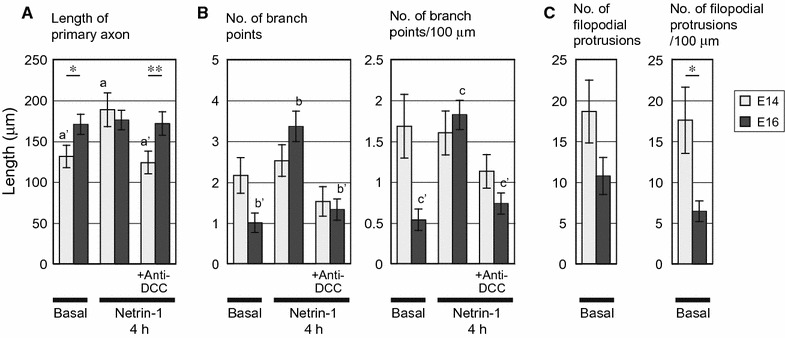
Quantitative morphometric analysis of outgrowth, branching, and filopodial protrusion in E14 and E16 cortical axons (**A**, **B**). Netrin-1-induced outgrowth of E14 axons and branching of E16 axons. The numbers of branch points (with branches longer than 12 μm and/or lamellipodium-tipped) were counted along the whole length of each primary axon. Error bars indicate the SEM (n = 25 from 3 experiments in E14 neurons, and from 4 experiments in E16 neurons). *a*, *b* and *c*: significantly higher than *a*′, *b*′ and *c*′, respectively, by Steel–Dwass test which was employed for comparisons among 3 test groups, within either E14 or E16 axons (p < 0.05). Besides, the length of primary axon (**A**) as well as the numbers and density of branch points (**B**) were compared between E14 and E16 axons: *p < 0.05 and **p < 0.01 by Wilcoxon rank sum test, which was employed for comparisons between E14 and E16 axons subjected to the same treatment out of the three (that is, under basal conditions or after 4 h netrin-1 stimulation in the absence or presence of an anti-DCC antibody). **C** Abundance of protrusions on the shafts of mouse E14 cortical axons under basal conditions. The numbers of filopodial protrusions (12 μm or shorter and lacking a lamellipodial tip) were counted along the whole length of each primary axon. Data were obtained from the sets of axons under basal conditions analyzed in **A** and **B** (25 per test group), and the numbers and densities of protrusions were compared between E14 and E16 axons. Error bars indicate the SEM. *p < 0.05 by Wilcoxon rank sum test

Next, we examined whether netrin-1-induced axon outgrowth and/or branching would occur in each group. After 5 days in vitro (DIV), E14 (Fig. 2) and E16 (Fig. 3) cortical neurons were subjected to netrin-1 treatment for 4 h [11, 15] in the presence or absence of an anti-DCC function-blocking antibody, and the lengths of primary axon shafts and the number of branch points were measured (Fig. 4A, B). In E14 neurons, netrin-1 increased the average length of primary axons but, in contrast, caused no change in the number and density of branches on them. This netrin-1-induced outgrowth was abolished by bath application of an anti-DCC antibody (Fig. 4A). These results indicated a significant contribution of the receptor DCC in promoting outgrowth of cortical axons, which is in accordance with previous reports in commissural axons [2, 4].

Contrary to the results obtained from E14 neurons, netrin-1 induced no significant change in the length of primary axons in E16 neurons (Fig. 4A). Netrin-1 increased both the number and density of branches on the primary axons, indicating that netrin-1 promoted formation of axon branches in E16 neurons (Fig. 4B). This netrin-1-induced axon branching was attenuated in the presence of an anti-DCC antibody (Fig. 4B), suggesting a substantial contribution of the receptor in promoting axon branching.

Comparisons between E14 and E16 axons in terms of these measurements are also demonstrated in Fig. 4A, B. A significant difference in axon length was observed between E14 and E16 axons under basal conditions. The difference was abolished after treatment with netrin-1, and observed again under the coexistence of an anti-DCC antibody with netrin-1.

In addition, the primary axons with one or more branches were subjected to the correlation analysis between axon length and the density of branches, employing Spearman’s rank correlation coefficient (ρ). A significant negative correlation was found in E14 axons under basal conditions (ρ = −0.735; n = 16; p = 0.0012), as well as in E14 axons with 4 h netrin-1 treatment in the absence of anti-DCC antibody (ρ = −0.527; n = 21; p = 0.014), indicating that longer axons had lower density of branches in these two experimental groups. No significant correlation was found in the other four groups, that is, E14 axons with netrin-1 treatment in the presence of the antibody (ρ = −0.261; n = 18), as well as E16 axons under basal conditions (ρ = −0.349; n = 14) and with netrin-1 treatment in the absence (ρ = 0.170; n = 23) or presence (ρ = −0.103; n = 17) of the antibody, with p values larger than 0.05.

### Effects of netrin-1 on the sprouting of filopodial protrusions from the cortical axon shafts

**Fig. 5:**
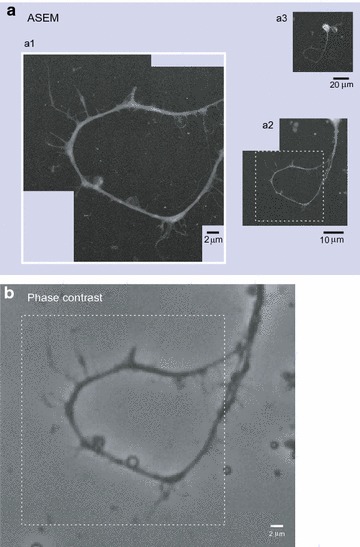
ASEM imaging of netrin-1-induced filopodial protrusions in comparison to phase contrast imaging. Imaging of an E16 cortical neuron treated with netrin-1 for 30 min was carried out by ASEM (**a**) and by phase contrast microscopy (**b **). Original magnifications of ASEM images are 6000× (*a1*), 2000× (*a2*), and 370× (*a3*). The rectangles with broken lines in (*a2*) and (**b**) indicate the area shown in (*a1*) with white frames

**Fig. 6:**
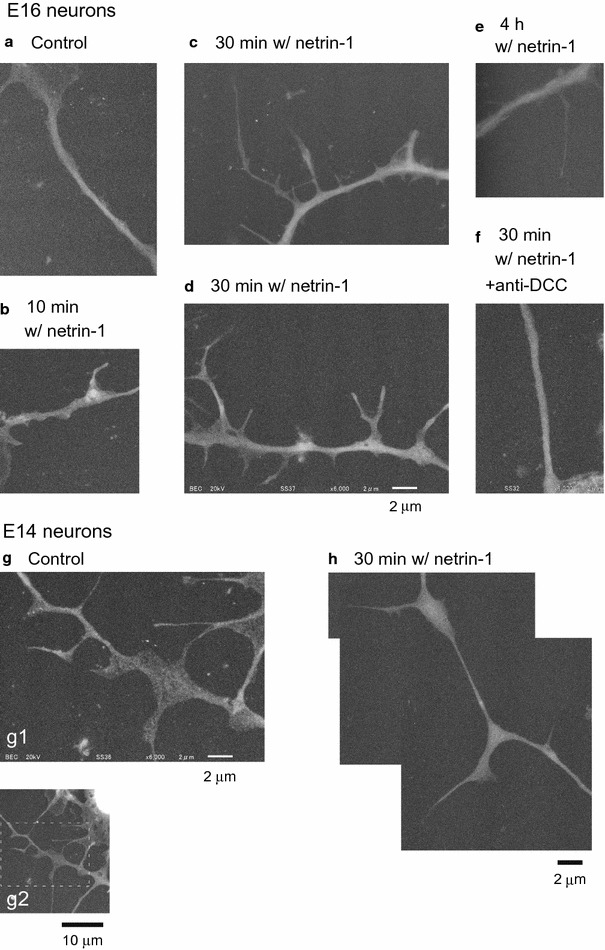
Visualization of filopodial protrusions on the axon shafts by ASEM. **a** An ASEM image of an E16 cortical axon under basal conditions (a negative control). (**b**–**e**) ASEM images of E16 axons treated with netrin-1 for 10 min (**b**), 30 min (**c**, **d**) and 4 h (**e**), showing netrin-1-induced filopodial protrusions. The image presented in **c** and the images presented in Fig. 5 are of the same neuron. **f** An ASEM image of an E16 axon treated with netrin-1 for 30 min in the presence of an anti-DCC function-blocking antibody. Few protrusions were found on the axon shaft. **g** ASEM images of E14 cortical culture under basal conditions, showing protrusions on the shaft of an axon. The rectangle with broken lines in (*g2*) indicates the area shown in *g1*. **h** A set of ASEM images of an E14 axon treated with netrin-1 for 30 min. Original magnifications are 6000× (**a**–**f**, *g1*, **h**) and 2000× (*g2*)

**Fig. 7:**
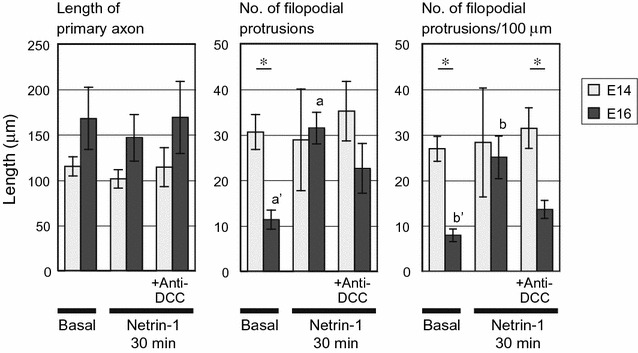
Netrin-1-dependent filopodial protrusions in E16 axons and netrin-1-independent protrusions on the shafts of E14 axons. Netrin-1 was bath-applied to cortical cultures at a concentration of 250 ng/mL for 30 min, with or without 1 μg/mL anti-DCC antibody applied 30 min before the addition of netrin-1. The numbers of filopodial protrusions (12 μm or shorter and lacking a lamellipodial tip) were counted along the whole length of each primary axon. Error bars indicate the SEM (n = 7). *a* and *b*: significantly higher than *a*′ and *b*′, respectively, by Steel–Dwass test employed for comparisons among 3 test groups (that is, under basal conditions and after 30 min netrin-1 stimulation in the absence or presence of an anti-DCC antibody), within either E14 or E16 axons (p < 0.05). *p < 0.01 by Wilcoxon rank sum test, which was employed for comparisons between E14 and E16 axons subjected to the same treatment

We then sought filopodial protrusions sprouted from the axon shafts of E16 neurons prior to axon collateral branching in response to netrin-1 employing ASEM (Figs. 5, 6), as well as employing differential interference contrast (DIC) microscopy followed by morphometric analysis (Fig. 7). In our previous study using cortical neurons of neonatal hamsters, an increase in filopodial protrusions was observed after 30 min netrin-1 treatment, and an increase in axon branching after 4 h treatment [11]. These time points were therefore included within the test groups in the present study.

ASEM images of E16 neurons are presented in Figs. 5 and 6a–f. Increased filopodial protrusions were observed on the axon shafts after treatment with netrin-1 (Figs. 5, 6b–e), in particular, after that for 30 min (Figs. 5, 6c, d), while few protrusions were found on the shafts under basal conditions (Fig. 6a). Few protrusions were likewise observed on the shafts of E16 axons when the netrin-1 treatment was carried out in the presence of an anti-DCC antibody (Fig. 6f).

Figure 7 shows the results of quantitative analysis of filopodial protrusions employing DIC images. The lengths of primary axon shafts and the number of filopodial protrusions were examined after 30 min netrin-1 treatment in the presence or absence of an anti-DCC antibody. In E16 neurons, netrin-1 induced an acute increase both in the number and density of filopodial protrusions on the shaft of primary axons.

As it was not known whether netrin-1 would promote sprouting of filopodial protrusions on the axon shafts concomitant with induction of axon outgrowth, we also examined protrusions in E14 axons by means of ASEM (Fig. 6g, h) as well as morphometric analysis employing DIC microscopy (Figs. 4C, 7); the results presented in Fig. 4C were obtained by analyzing the specimens of neurons under basal conditions employed in Fig. 4A, B. In the morphometric analysis, the density of protrusions in E14 axons was significantly higher than that in E16 axons under basal conditions (Figs. 4C, 7). These analyses collectively revealed that a substantial portion of E14 neurons had an abundance of protrusions on their axon shafts, irrespective of netrin-1 treatment.

## Discussion

A variety of physiological roles are known for an axon guidance cue netrin-1. In this study, netrin-1-induced axon outgrowth was observed in cerebral cortical neurons dissociated from E14 mice, while netrin-1-induced axon branching was observed in E16 cortical neurons. Netrin-1-induced outgrowth of mouse cerebral cortical axons was reported in vivo, which accounts for a few steps in the development of corticofugal tracts including their extension toward, and then into, the internal capsule in embryonic mice starting from E12.5 [7, 17, 22, 24]. Moreover, Métin et al. reported outgrowth of mouse cortical axons using E12.5 explants cultured for 18 h in the presence of netrin-1 [7]. Our observation in mouse E14 neurons grown for 5 DIV (Figs. 2, 4A, B) is in accordance with these previous reports.

On the other hand, we and others previously reported netrin-1-induced axon branching without outgrowth of primary axons in neuronal culture dissociated from sensorimotor cortices of neonatal Syrian golden hamsters, of which gestation period is as short as 16 days [8–11]. At this stage the axons of the cultured neurons were reported to develop interstitial branches similar in numbers, location, and time course to those in vivo [8, 30]. In the present study, netrin-1-induced axon branching was observed in mouse E16 cortical neurons grown for 5 DIV (gestation period of mice: 19-to-20 days), which had similarities to that observed previously in hamster P0-P3 neurons grown for 5–7 DIV [11] in the following two aspects. That is, (1) both of them were not accompanied by outgrowth of primary axons (Fig. 4A, B), and (2) both involved, and were thought to originate from sprouting of filopodial protrusions induced by netrin-1 on the axon shafts; indeed, filopodial protrusions were found in abundance by ASEM, on the shafts of mouse E16 axons after 30 min netrin-1 treatment (Figs. 5, 6c, d), and morphometric analysis verified an increase in the number of the protrusions (Fig. 7).

There remained an issue whether netrin-1 would stimulate sprouting of filopodial protrusions from the shafts when it promotes axon outgrowth, as was the case in promoting collateral branching. What observed in E14 axons, however, was an abundance of protrusions irrespective of netrin-1 treatment: both phase contrast microscopy and ASEM unexpectedly showed that a substantial portion of E14 cortical axon shafts had lots of protrusions even in the absence of netrin-1 (Figs. 2b, 6g); morphometric analysis revealed that the density of protrusions in E14 axons was significantly higher than that in E16 axons under basal conditions (Figs. 4C, 7), although the measured values of the density in E14 axons varied between these two experiments. We cannot rule out the possibility that some portion of filopodial protrusions might be too small to be detected at the magnification of DIC microscopy adopted herein, which could potentially cause variance in the results. These netrin-1-independent protrusions in E14 axons are yet to be characterized; therefore, further study is needed to find out whether/how they are relevant to the netrin-1-dependent filopodial protrusions in E16 axons.

As compared to E16 axons, E14 axons exhibited not only high density of netrin-1-independent pre-existing filopodial protrusions (Figs. 4C, 7) but also high density of spontaneously developed axon branches under basal conditions (Fig. 4B), suggesting the possibility that the short axon length is a factor in these high densities in E14 neurons. Notably, the analysis of correlation between axon length and the density of branches showed that longer axons had lower density of branches in E14 axons with and without 4 h netrin-1 treatment. Therefore, although the average value of branch density was hardly affected by this treatment (Fig. 4B), it is still possible in E14 axons that netrin-1-dependent axon outgrowth, resulting in an increase in the axon length, is a factor in decreasing the density of branches.

Our results presented here provide an explanation as to why both axon outgrowth and branching have been reported in rodent cortical axons in response to netrin-1. The results, while each of the E14 and E16 culture was maintained for an additional 5-day period in vitro, suggested a switch in netrin-1 function in cortical neurons during development, from promotion of axon outgrowth to promotion of axon branching. Since the neurons in different cortical layers are born and differentiate at different times, the culture employed here should be highly heterogeneous containing multiple populations of neurons originated from different layers of each area in the cerebral cortex [15]. Previous studies indicate that multiple populations of cerebral cortical neurons exist: two subpopulations, with different axon elongation rates and distinct responses to netrin-1, were found in dissociated cortical neurons of E14 mice by microfluidic compartmentalization experiments [31]; layer-dependent expression of netrin-4 was reported in sensory cortices of postnatal rats, which was suggested to be responsible for terminal branching of thalamocortical axons that occurs specifically in layer 4, the target layer [32]. Therefore, it was thought that a population of neurons showing axon outgrowth in response to netrin-1 was dominant in E14 culture, whereas a population showing axon branching became dominant in E16 culture, although it is not clear whether these two populations have the same origin or discrete origins. If the former is the case, it follows that our observation implies a switch in the responsiveness of cortical axons to netrin-1, from growth cone attraction to axon collateral branching.

While sprouting of actin filament-based filopodial protrusions is thought to be an initial step of axon collateral branching, invasion of axonal microtubules into the protrusions is required for further development and stabilization of nascent axon branches, without being retracted back into the axons [16, 17]. As the dynamics and organization of axonal microtubules is regulated by factors such as microtubule-associated proteins, these factors might be involved in switching of netrin-1 function from promotion of axon outgrowth to promotion of axon branching, by modulating the effects of netrin-1 on axonal microtubules.

Expression of DCC protein was observed both in E14 and E16 cortical axons of mice (Fig. 1), and bath application of a function-blocking antibody revealed significant DCC contribution not only to netrin-1-induced axon outgrowth as previously known [2, 4], but also to netrin-1-induced cortical axon branching. It will be interesting to find out whether/how distribution of DCC would be implicated in the exertion of these netrin-1 functions. In our previous study, we examined the changes in DCC distribution concomitant with netrin-1-induced axon branching in primary cortical neurons of hamster neonates, using the same antibody against DCC as that we used in the present study [11]. In that paper, we reported netrin-1-induced cluster formation of DCC at the surface of axon shafts via exocytosis; no preference was detected in the sites of the cluster formation, as DCC clusters were apparently scattered randomly over the surface of axon shafts. Therefore, we have been thinking of the possibility that netrin-1, instead of determining the location of filopodia and/or axon branches, raises the probability of filopodial protrusion and subsequent axon branching along the whole length of each axon shaft. Further study, however, is needed to evaluate this possibility.

Contribution of DCC-mediated netrin signaling to axon arborization (and to synaptogenesis, which is known to be closely related to axon arborization) was reported in retinal ganglion cells of *Xenopus laevis* during retinotectal development [29]. More recently, netrin-1-induced axon branching was reported also in cerebral cortical neurons prepared from embryonic mice, in E15.5 neurons grown for 3 DIV [13] and E15 neurons grown for 90 h in vitro [12]; the former paper suggested that a DCC binding protein TRIM9 (tripartite motif-containing protein 9) E3 ubiquitin ligase regulates netrin-1-induced axon branching via its interaction with SNAP25 (synaptosomal-associated protein 25), which is netrin-1-sensitive and constrains exocytosis; the latter suggested that binding of DCC and another netrin receptor DSCAM to a dynamic β-tubulin isoform TUBB3, regulates microtubule dynamics in netrin-1-induced axon branching. Our results presented here, together with others, raised the possibility that netrin-1/DCC signaling could be involved in both of the axon outgrowth and axon collateral branching, although the results do not exclude the possibility of contribution by other netrin receptors. The factor(s), presumably intrinsic one(s), determining which of these functions netrin-1 exerts is(are) yet to be identified.

It is believed that axon branching is modulated independently of axon outgrowth/attraction [10, 14, 15]. It has been reported, in cortical neurons prepared from neonatal hamsters, that both CaMKII (calcium/calmodulin-dependent protein kinase II) and MAPK (mitogen-activated protein kinase) are required for netrin-1-induced axon branching, while CaMKII, but not MAPK, is involved in netrin-1-induced axon outgrowth [9]. Notably, axon outgrowth and collateral branching differ in their locations of occurrences in neuronal cells: the former occurs in the growth cones, while the latter in the axon shafts. Correct axon guidance in developing corticofugal tracts implicates combinatorial expression of transcription factors that is specific to each subtype of cortical neurons in a certain developmental stage [22, 24]. To identify what accounts for the difference between netrin-1-induced outgrowth and branching in cerebral cortical axons, to elucidate its molecular basis, and eventually, to understand the mechanism that underlies the shift in the function of netrin-1 are issues yet to be solved.

## Conclusions

We showed two distinct functions of netrin-1—promotion of axon outgrowth, and promotion of axon collateral branching that involves filopodial protrusion from the shafts—in mouse cerebral cortical neurons in vitro. As netrin-1 promoted outgrowth in E14 axons and branching in E16 axons, and thus exhibited each function depending on the stage of embryos utilized for cortical culture, our study raised the possibility of a shift in netrin-1 function during cortical development. Furthermore, our results suggested that a netrin-1 receptor DCC may contribute to both cortical axon outgrowth and branching.

## Methods

### Reagents

All reagents were purchased from Sigma (St. Louis, MO) unless otherwise specified. A mouse monoclonal antibody raised against the extracellular domain of human DCC protein (clone AF5) was purchased from Millipore (Billerica, MA; Cat# OP45; RRID: AB_2292666) and utilized not only for detecting DCC protein but also as a function-blocking antibody against DCC [2, 6, 33]. A rat monoclonal antibody against mouse L1 protein (clone 324) was purchased from Millipore (Cat# MAB5272; RRID: AB_2133200).

### Dissociation of mouse cerebral cortical neurons for cell culture

All animal experiments were approved by the Institutional Animal Care and Use Committee of Saitama Medical University.

Timed-pregnant C57BL/6 N mice were purchased from Japan SLC, Inc. (Shizuoka, Japan). Dissociated cultures were prepared from cerebral cortex of mice on E14 and E16, essentially by the method of Dent and Kalil [19] with minor modification; E0 was defined as the day of vaginal plug detection. The cerebral cortex was dissected under a dissection microscope from the embryonic brain in ice-cold Hanks’ balanced salt solution (HBSS) (Lonza, Basel, Switzerland) with B27 supplement (Gibco, Grand Island, NY) and then cut into small pieces. Cortical pieces were digested with 0.025% trypsin/EDTA (Gibco) and 0.05% DNase I in HBSS without Ca^2+^/Mg^2+^ (Gibco) for 15 min at 37 °C, and dissociated by trituration in plating medium (neurobasal medium (Gibco), 5% fetal bovine serum (Nichirei Biosciences Inc., Tokyo, Japan), B27 supplement, 0.3% glucose, 1 mM l-glutamine (Gibco), and 37.5 mM NaCl). Cell suspension was filtrated with a cell strainer of 70 μm mesh size (BD Falcon, Bedford, MA) and centrifuged at 500 rpm (46×*g*) for 7 min.

Cells obtained were resuspended in plating medium and utilized for 5 d culture on coverslips or dishes treated in advance with poly-d-lysine (1 mg/mL in borate buffer, pH 8.5) for 1 h; laminin-1 was excluded from the substrate in this study, since Höpker et al. reported that it converts netrin-1-mediated attraction into repulsion in *Xenopus* retinal growth cones [34]. To perform immunocytochemical and morphometric analyses, dissociated cells were plated on coverslips at a density of 60,000 cells/cm^2^; after 1 h, medium was changed to a serum-free formulation (neurobasal medium, B27 supplement, 0.3% glucose, 1 mM l-glutamine, and 37.5 mM NaCl); cultures were then maintained at 37 °C in 5% CO_2_ for 5 DIV until treated as described in the next subsection. To perform ASEM, specialized dishes were employed for culture and cells were treated as described later.

### Immunocytochemistry and optical microscopy

For immunocytochemical analysis of DCC, cells on coverslips were fixed with 4% paraformaldehyde (Wako, Osaka, Japan) in Krebs’ buffer with 0.4 M sucrose for 20 min at room temperature [15]. Fixed cells were treated with 0.1% Triton X-100 for 5 min, and then incubated with an anti-DCC antibody (1 μg/mL) for 1 h. DyLight 488-conjugated donkey anti-mouse IgG (H + L) (Jackson ImmunoResearch, West Grove, PA) was used at 17.5 μg/mL to visualize total DCC. After mounting the coverslips in a drop of ProLong Gold mounting medium (Thermo Fisher Scientific Inc., Waltham, MA), twelve-bit grayscale images of neurons were collected using an inverted fluorescence microscope (model TE2000-U; Nikon, Tokyo, Japan) equipped with 60×/1.45 NA oil-immersion and 40×/0.75 NA dry objectives, and a cooled charge-coupled device (CCD) camera (model ORCA C4742-95-12NR; Hamamatsu Photonics, Shizuoka, Japan).

For morphometric analysis, cortical cultures were treated with 250 ng/mL netrin-1 (R&D Systems, Minneapolis, MN) for 30 min or 4 h by bath application [11, 15] in the presence or absence of 1 μg/mL anti-DCC function-blocking antibody that was bath-applied 30 min earlier than netrin-1 application [33], then fixed. In the initial several rounds of the experiments, surface L1 was visualized to distinguish the axons from the dendrites, since L1 is known to accumulate at the axonal surface [35]; for this purpose, fixed cells were incubated with an anti-L1 antibody (5 μg/mL) for 16 h without permeabilization, and then incubated with Cy3-conjugated donkey anti-rat IgG (H + L) (15 μg/mL; Jackson ImmunoResearch) for 1 h. After collecting DIC and phase contrast images (as well as epifluorescence images in case of surface L1 immunocytochemistry), we employed DIC images for morphometric analysis.

### Data analysis

DIC images were analyzed morphometrically employing MetaMorph 7.6 software (Molecular Devices, Sunnyvale, CA). E14 and E16 neurons after 4 h treatment with or without netrin-1 were subjected to the analysis of axon branching/outgrowth [11]: the lengths of primary axon shafts (excluding growth cones) were measured and the numbers of branch points (with branches longer than 12 μm and/or lamellipodium-tipped, [11]) were counted along the whole length of each primary axon. E14 and E16 neurons after 30 min treatment were employed for the analysis of filopodial protrusion on the axon shafts [11]: in addition to the measurement of the lengths of primary axon shafts, the numbers of filopodial protrusions (12 μm or shorter and lacking a lamellipodial tip) were counted along each primary axon. Following Jarque–Bera test for normality, nonparametric statistical analyses were performed on the data: Steel–Dwass test or Wilcoxon rank sum test was employed where appropriate. Correlation between axon length and the density of branches was analyzed using Spearman’s rank correlation test, which was applied for the primary axons with at least one branch. Differences were considered significant if p < 0.05.

### ASEM

ASEM, a recently developed technique [25], was performed to visualize filopodial protrusions on the axon shaft with resolution of electron microscope, but with reduced artifacts in comparison to conventional electron microscopy. A specimen for ASEM must be set in the specialized 35-mm sample dish (ASEM dish; JEOL Ltd., Tokyo, Japan), which is equipped with an electron-permeable silicon nitride (SiN) film window (100 nm thick, 0.25 × 0.25 mm^2^ wide) in its base. During image acquisition employing the ClairScope system (model JASM-6200; JEOL), the electron beam of inverted SEM scans the sample in solution through the window which separates vacuum (below) and atmosphere (above), from underneath. Backscattered electrons are captured by the backscattered electron imaging (BEI) detector located just beneath the window.

In this study, cortical neurons were cultured on the ASEM dish. In advance of plating cells, poly-d-lysine coating was applied to the SiN film window using 40 μL of 1 mg/mL solution. Twenty microliters of cell suspension containing 15,000 cells in plating medium was used for inoculation on the coated SiN film window. After 1 h incubation at 37 °C in 5% CO_2_, 2 mL of serum free medium was added to the culture. Cultures were maintained for 5 DIV and then treated with 250 ng/mL netrin-1 for 10 min, 30 min and 4 h, with or without an anti-DCC function-blocking antibody (1 μg/mL) treatment starting 30 min earlier than netrin-1 application. Cultures were then fixed with 2.5% glutaraldehyde (TAAB Laboratories Equipment Ltd., Berks, England) in 0.1 M phosphate buffer, pH 7.4 for 30 min and washed three times with double distilled water (DDW). Fixed cells were stained with 2% phosphotungstic acid [25, 36] for 30 min and washed three times with DDW. Specimens within the ASEM dishes were then directly imaged in DDW supplemented with a radical scavenger, either dextrose (10 mg/mL) [37, 38] or propyl gallate (saturated solution) [39], employing the ClairScope system.

In order to compare with ASEM images, phase contrast images of specimens on the ASEM dishes were acquired employing the BIOREVO all-in-one inverted microscope system (model BZ-9000; Keyence, Osaka, Japan) equipped with a 40×/0.60 NA objective.

## Abbreviations

ASEM: atmospheric scanning electron microscopy
BEI: backscattered electron imaging
CaMKII: calcium/calmodulin-dependent protein kinase II
CCD: cooled charge-coupled device
DCC: deleted in colorectal cancer
DDW: double distilled water
DIC: differential interference contrast
DIV: days in vitro
DSCAM: Down syndrome cell adhesion molecule
E: embryonic day
HBSS: Hanks’ balanced salt solution
MAPK: mitogen-activated protein kinase
P: postnatal day
SiN: silicon nitride
SNAP25: synaptosomal-associated protein 25
TRIM9: tripartite motif-containing protein 9
